# Loss or inhibition of lysosomal acid lipase *in vitro* leads to cholesteryl ester accumulation without affecting muscle formation or mitochondrial function

**DOI:** 10.1016/j.bbadva.2024.100135

**Published:** 2024-12-25

**Authors:** Alena Akhmetshina, Laszlo Schooltink, Melina Amor, Katharina B. Kuentzel, Silvia Rainer, Ananya Nandy, Hansjoerg Habisch, Tobias Madl, Elizabeth Rendina-Ruedy, Katharina Leithner, Nemanja Vujić, Dagmar Kratky

**Affiliations:** a Gottfried Schatz Research Center, Molecular Biology and Biochemistry, Medical University of Graz, Graz, Austria; b Division of Clinical Pharmacology, Department of Medicine, Vanderbilt University Medical Center, Nashville, TN, USA; c Otto Loewi Research Center, Division of Medicinal Chemistry, Medical University of Graz, Graz, Austria; d BioTechMed-Graz, Graz, Austria; e Molecular Physiology and Biophysics, Vanderbilt University, Nashville, TN, USA; f Division of Pulmonology, Department of Internal Medicine, Medical University of Graz, Graz, Austria

**Keywords:** LAL, LAL deficiency, Energy metabolism, Primary myoblast, Skeletal muscle, Myogenesis, C2C12 cells

## Abstract

Skeletal muscle (SM) is essential for movement, stability, and overall body function, and it readily adapts to changes in energy demand. Myogenesis is energy-intensive and involves complex molecular and cellular events. We recently demonstrated that the absence of lysosomal acid lipase (LAL) *in vivo* significantly impacts the SM phenotype, primarily by disrupting energy homeostasis and reducing ATP production. As systemic LAL deficiency affects multiple organs, we hypothesized that the altered SM phenotype resulted from systemic rather than SM-specific loss of LAL activity.

To distinguish between systemic and cell-intrinsic effects, we used primary myoblasts isolated from Lal-deficient (-/-) mice as well as C2C12 cells treated with the pharmacological inhibitor of LAL, Lalistat-2. We found a significant accumulation of cholesteryl esters in both models studied, highlighting the central role of LAL in lipid catabolism in the SM. However, lipid accumulation was absent under lipoprotein-deficient culture conditions. Neither genetic loss nor pharmacological inhibition of LAL affected myofiber formation or mitochondrial function *in vitro*, in contrast to what we observed in SM isolated from Lal-/- mice. Tracing [^13^C_6_]-labeled glucose in both cell culture models revealed only minor changes in tricarboxylic acid cycle metabolites. These results suggest that although LAL plays an essential role in lipid metabolism, its impact on the processes involved in muscle differentiation and cellular energy production is minor. We conclude that the cell-intrinsic effects of Lal-/- SM are unlikely to drive the SM phenotype observed *in vivo*.

## Highlights

• LAL deficiency leads to cholesteryl ester accumulation in skeletal muscle cells.
• Mitochondrial function of muscle cells is unaffected by LAL loss/inhibition.
• Muscle cell formation and differentiation are unaffected by LAL loss/inhibition.
• Systemic rather than muscle-specific LAL deficiency affects muscle phenotype.

## Graphical abstract

**Figure:**
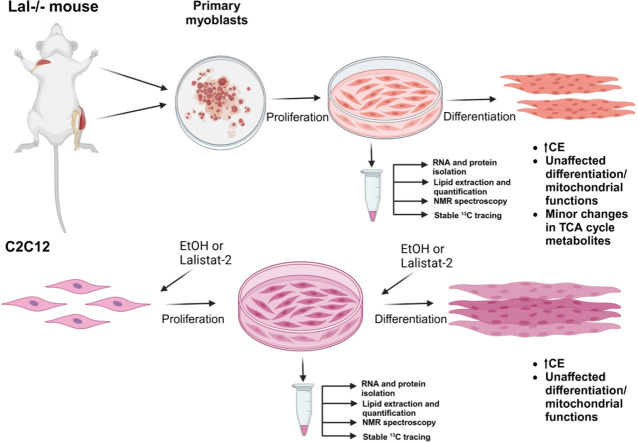

## 1 Introduction

Skeletal muscle (SM) is a dynamic tissue that plays a central role in maintaining the body's stability, facilitating movement, and ensuring overall functionality [1]. To function properly, SM has a high energy demand that is met by its ability to adapt its energy substrates in response to environmental conditions [2]. One of the most energy-demanding processes is myotube formation during myogenesis, which is orchestrated by a complex interplay of molecular and cellular events. During the early stages of embryogenesis, myoblasts either proliferate or differentiate into myotubes. *In vitro* studies have shown that myoblasts tend to proliferate when sufficient growth factors are available, whereas they differentiate into myotubes when growth factors are limited [3]. During differentiation, myoblasts undergo morphological and biochemical changes, including alignment, elongation, fusion into multinucleated myotubes, and expression of muscle-specific proteins such as myosin and actin [4].

We have recently demonstrated that the systemic loss of lysosomal acid lipase (LAL) profoundly affects the SM phenotype in mice [5]. LAL plays a vital role in lysosomes [6,7] by catalyzing the breakdown of neutral lipids, including cholesteryl esters (CE) and triacylglycerols (TG) into free cholesterol (FC) and fatty acids, respectively [8]. Therefore, LAL-mediated lipid degradation affects cellular energy and cholesterol homeostasis, and its lipolytic products regulate catabolic, anabolic, and signaling pathways [7]. Mutations in the LAL-encoding *LIPA* gene lead to early-onset or late-onset LAL deficiency (LAL-D), a rare autosomal-recessive lysosomal storage disorder that leads to early death in severly affected patients (reviewed in [9]). In a LAL-D mouse model, we observed a pronounced SM fiber type switch and altered SM functions as a result of reduced ATP production due to dysfunctional mitochondria and impaired energy metabolism [5]. However, LAL-D is a serious disease that affects multiple tissues, including the liver [10,11], small intestine [[12], [13], [14]], and adipose tissue [11,15], leading to a massive macrophage infiltration in metabolically active tissues and elevated systemic inflammation [[11], [12], [13], [14],^16^]. Consequently, it remained unclear whether the observed reduction in muscle mass, fiber type switch, and increased muscle fatigue were attributable to the loss of LAL activity solely in SM.

Here, we therefore aimed to discriminate whether the changes we observed in the SM of global Lal-/- mice were caused by a systemic or SM-specific loss of LAL activity. Since LAL is a secreted protein, muscle cell-specific Lal-deficient mice may not exhibit a phenotype attributable to the uptake of LAL by myoblasts. We therefore investigated the effects of defective LAL activity in the SM by using primary myoblasts isolated from Lal-/- mice as well as C2C12 cells, a widely used model for studying the development, regeneration, and differentiation of myoblasts into mature muscle fibers (myotubes) *in vitro* [17]. To reproduce the conditions of LAL-D in C2C12 cells, we treated them with Lalistat-2, a pharmacological inhibitor of LAL [[18], [19], [20]]. In both of our cell models, loss of LAL activity led to increased CE concentrations while maintaining myofiber formation. Unaltered mitochondrial function *in vitro* suggested that the differences between *in vivo* and *in vitro* models may be linked to nutrient availability and systemic inflammation in Lal-/- mice.

## 2 Materials and methods

### 2.1 Primary myoblast isolation

Male and female 10- to 16-week-old Lal-/- mice and their corresponding wild-type littermates on a C57BL/6J background were euthanized, hindlimb SM were harvested in a Petri dish containing 0.2% bovine serum albumin (BSA) and 0.5% penicillin/streptomycin in Hanks' balanced salt solution, washed, and then minced in a digestion buffer (3 mg/mL dispase II, 10 µg/mL DNAse I, 2 mg/mL collagenase A (all Roche, Basel, Switzerland), 8 mM CaCl_2_, and 5 mM MgCl_2_). Digestion was performed for 45–60 min at 37 °C, with resuspension and stirring at regular intervals. Afterwards, the cells were filtered through a 40-µm^2^ pore sieve, centrifuged, and treated with ammonium-chloride-potassium lysis buffer. Freshly isolated cells were plated with several pre-plating steps at a high density on gelatin-coated dishes in growth medium containing high-glucose Dulbecco's Modified Eagle's Medium (DMEM, 25 mM glucose), 20% FBS, 10% horse serum, 1% HEPES, 1% sodium pyruvate, 1% penicillin/streptomycin, and 5 ng/mL fibroblast growth factor (all Thermo Fisher Scientific, Waltham, MA). After 3–5 days, the medium was replaced with a differentiation medium, consisting of DMEM, 10% horse serum, and 1% penicillin/streptomycin, for a period of 3 days.

### 2.2 Culture conditions for C2C12 cells

The immortalized mouse myoblast C2C12 cell line was cultured in high-glucose DMEM, which was supplemented with 10% FBS or 10% lipoprotein-deficient serum (LPDS) and 1% penicillin/streptomycin at 37 °C and 5% CO_2_. For differentiation, C2C12 cells were incubated with DMEM supplemented with 2% horse serum or 10% LPDS and 1% penicillin/streptomycin. To inhibit LAL activity, cells were cultured for 4 days (proliferation experiment) and 3 or 6 days (differentiation experiment) with 0.1, 1, or 10 µM Lalistat-2 (Sigma-Aldrich, St. Louis, MO) dissolved in ethanol (EtOH; final concentration: 0.1%).

### 2.3 Cholesteryl ester and triacylglycerol hydrolase activity assays

Acid and neutral CE hydrolase (CEH) and TG hydrolase (TGH) activities were measured in C2C12 cell lysates as previously described [21]. Briefly, cells were lysed in a citrate (pH 4.2) or phosphate lysis buffer (pH 7), sonicated 2 × 10 s on ice and centrifuged at 1000 *x g* at 4 °C for 10 min. Enzyme activities were measured in the supernatant. The substrate for measuring CEH activity contained 200 µM cholesteryl oleate/sample, 0.04 µCi/sample cholesteryl [1–^14^C]-oleate (Amersham Biosciences, Amersham, UK), and 455 µM mixed micelles of phosphatidylcholine and phosphatidylinositol (3:1) (Amersham Biosciences). The substrate for the TGH activity assay contained 300 µM triolein/sample, 0.5 µCi/sample [9,10–^3^H(N)]-triolein (Perkin Elmer, Waltham, MA), and 45 µM of above-mentioned mixed micelles.

The samples were incubated in a water bath for 1 h at 37 °C under constant shaking. The reaction was terminated by the addition of 3.25 mL of stop solution (methanol/chloroform/n-heptane, 10:9:7, v/v/v) and 1 mL of 0.1 M potassium carbonate (pH 10.5). After vortexing and centrifugation at 800 *x g* for 15 min at 4 °C, the radioactivity in 1 mL of the upper phase was quantified by liquid scintillation counting and the release of fatty acids was calculated as described [22].

### 2.4 Cell viability measurement by the MTT assay

The 3-[4,5-dimethylthiazol-2-yl]-2,5-diphenyltetrazolium bromide (MTT) assay was employed to quantify cellular metabolic activity as an indicator of cell viability. To this end, C2C12 cells were washed with PBS and incubated with a 100 µM MTT (stock solution 5 mg/mL MTT in PBS, pH 7.4; Sigma-Aldrich, St. Louis, MO) in 1 mL of culture medium for 2 h at 37 °C. Subsequently, the cells were lysed with 40 mM HCl in isopropanol for 10 min on a shaker to dissolve the formazan crystals. The absorbance of the blue formazan in the cell lysate was measured spectrophotometrically at 570 nm using a CLARIOstar® microplate reader (BMG Labtech, Ortenberg, Germany), with a reference wavelength of 650 nm.

### 2.5 Doubling assay

A total of 10,000 C2C12 cells and 40,000 primary myoblasts were seeded in each well of a 6-well plate. The cells were stained with trypan blue (Thermo Fisher Scientific, Waltham, MA) and manually counted after 6, 24, and 72 h (C2C12) or 24, 72, and 96 h (primary myoblasts) using a hemocytometer.

### 2.6 RNA isolation, reverse transcription, and real-time PCR

Total RNA from C2C12 cells and primary myoblasts was extracted using the TRIsure™ reagent (Meridian, Memphis, TN) and the Monarch total RNA miniprep kit (New England Biolabs, Ipswich, MA), respectively, following the manufacturers' instructions. Subsequently, 1 µg of RNA was reverse transcribed using the High-Capacity cDNA Reverse Transcription Kit (Applied Biosystems, Carlsbad, CA). Real-time qPCR was performed using the CFX Duet Real-Time PCR System (Bio-Rad Laboratories, Hercules, CA). Gene expression levels were analyzed in duplicate using the 2^−ΔΔCt^ method and *cyclophilin A* as housekeeping gene. The primer sequences are listed in supplementary Table S1.

### 2.7 Western blotting

Cell lysates were sonicated twice for 10 s on ice in RIPA buffer (150 mM sodium chloride, 1% NP-40, 0.5% sodium deoxycholate, 0.1% SDS, 50 mM Tris, pH 8). Protein concentrations were determined using the DC protein assay (Bio-Rad Laboratories, Hercules, CA). Thereafter, 30 µg of protein was separated by SDS-PAGE and transferred to a PVDF membrane. The membrane was incubated overnight with a mouse anti-fast myosin skeletal heavy chain antibody against MyHCIIx (#ab51263, 1:500; Abcam, Cambridge, UK). α-Tubulin (NB100–690, 1:1000; Novus Biologicals, Centennial, CO) antibody was used as loading control. Anti- mouse (Dako, Glostrup, Denmark) secondary HRP-conjugated antibody was visualized using the Clarity^TM^ Western ECL Substrate Kit (Bio-Rad Laboratories) and the ChemiDoc MP imaging system (Bio-Rad Laboratories).

### 2.8 Immunofluorescence staining

C2C12 cells were seeded on glass slides, washed three times with 500 µL PBS, and fixed by incubation with 500 µL of 4% paraformaldehyde in PBS for 20 min at room temperature (RT). The cells were washed again with PBS and permeabilized with 0.1% Triton X-100 in PBS for 10 min at RT, followed by three washing steps with 0.1% Tween-20 in PBS (PBST), each for 10 min at RT. Thereafter, the cells were blocked with 1% BSA in PBST for 1 h at RT. The slides were incubated overnight at 4 °C with anti-MyHCIIx antibody (#ab51263, 1:300; Abcam, Cambridge, UK). Following four PBST washing steps, the slides were blocked with 1% BSA in PBST for 15 min at RT and then incubated with a secondary goat anti-rabbit Alexa Fluor-488 antibody (#A-11008, 1:250; Thermo Fisher Scientific, Waltham, MA) in 1% BSA in PBST for 1 h at RT. Subsequently, the cells were washed three times with PBST and then incubated with DAPI for 10 min to stain the nuclei. Finally, the cells were mounted using Dako fluorescence mounting medium (Agilent Technologies, Santa Clara, CA) for examination under an Olympus BX63 fluorescence microscope equipped with an Olympus DP73 camera (Olympus, Shinjuku, Japan). The Myotube Analyzer app (license: CC BY-NC 4.0) was used to calculate the fusion index.

### 2.9 Oil red O staining

C2C12 cells and primary myoblasts were seeded on glass slides, washed with PBS, and fixed with 4% formaldehyde in PBS for 20 min at RT. Thereafter, the cells were washed once more with PBS and stained with ORO working solution (0.5% ORO in isopropanol, mixed with ddH_2_O (3:2) for 10 min, filtered) for 1 h. The nuclei were stained for 10 min with Mayer's hematoxylin (Carl Roth, Karlsruhe, Germany) and blued under running tap water. Images were taken with an Olympus BX63 microscope equipped with an Olympus DP73 camera (Olympus, Shinjuku, Japan).

### 2.10 Lipid extraction and quantification

C2C12 cells and primary myoblasts were washed three times with ice-cold PBS. Lipids were extracted with 1 mL of ice-cold hexane:isopropanol (3:2, v:v) at 4 °C for 2 h, after which the extracts were mixed with 15 µL of 10% Triton X-100 (in isopropanol). The organic solvents were evaporated under a stream of nitrogen gas, and the lipids were re-dissolved in 150 µL of deionized water by vigorous mixing and incubation in a water bath at 45 °C for 40 min. Thereafter, the TG, TC, and FC concentrations were quantified using enzymatic kits following the manufacturer's instructions (DiaSys, Holzheim, Germany), in the presence of sodium 3,5-dichloro-2-hydroxy-benzenesulfonate serving as a reaction enhancer. CE concentrations were calculated by subtracting FC from TC and normalized to protein concentrations.

### 2.11 Sample preparation, data acquisition, and metabolomic analysis by nuclear magnetic resonance (NMR) spectroscopy

*Ad libitum* fed male Lal-/- mice and their corresponding wild-type littermates (12–17 weeks of age) on a C57BL/6J background were fused for the experiments. The mice were maintained in a clean, temperature-controlled (22 ± 1 °C) environment on a regular 12-h/12-h light-dark cycle. We analyzed gastrocnemius segments enriched either in oxidative or glycolytic fibers. The samples were snap-frozen and processed following the methods described previously [23]. The NMR spectra were processed and analyzed as recently described [24].

### 2.12 Stable ^13^C isotope tracing and gas chromatography - mass spectrometry (GC–MS)

Stable isotopic tracing and GC–MS were performed as described previously [25]. The primary myoblasts and C2C12 were differentiated for 4–5 days in DMEM with 10% of horse serum, supplemented with [^13^C_6_]-glucose (Cambridge Isotope Laboratories, Tewksbury, MA) 24 h prior the end of differentiation. Metabolite extraction, derivatization, and analysis were conducted in accordance with established protocols [26]. Briefly, the metabolites were extracted on ice using cold 62.5% methanol in water with norvaline as an internal standard, followed by the addition of chloroform and sonication. For the analysis of conditioned media, 2 µL of each sample were extracted. After centrifugation at 4 °C for 10 min, the phases were separated. The polar metabolites were then dried by vacuum centrifugation. For GC–MS analysis, the samples were derivatized with 20 mg/mL methoxyamine in pyridine for 1 h at 37 °C, followed by N-(tert-butyldimethylsilyl)-N-methyl-trifluoroacetamide (TBDMS) for 30 min at 60 °C.

The separation was performed using an Agilent 7890B GC system coupled to an Agilent 5977A Inert MS system with a DB35MS column and helium as the carrier gas. Samples were injected in splitless mode, and the GC oven was temperature-programmed from 100 °C to 300 °C. Mass spectrometry was conducted in electron ionization (EI) mode at 70 eV in the mass range m/z 100–605. Peaks were quantified using El-Maven software [27], and the distribution of isotopologues was corrected to natural abundance with IsoCor [28]. Regular standards were used for metabolite identification. Quantification was relative, with metabolite amounts normalized to the internal standard and total protein concentration.

### 2.13 Mitochondrial stress test by Seahorse flux analysis

C2C12 cells were plated at a density of 2000 cells/well in a 96-well Seahorse XFe microplate (Agilent, Santa Clara, CA) and cultured in differentiation media with 0.1 µM Lalistat-2 or 0.1% EtOH (control) for 6 days before being analyzed as described previously [29]. Briefly, prior to the assay, the cells were washed with basal assay medium (Agilent) in the presence of 10 mM glucose, 2 mM glutamine, 1 mM sodium pyruvate, 200 nM insulin, and 60 µM oleic acid-BSA (all Sigma-Aldrich, St. Louis, MO). A mitochondrial stress test was performed through the sequential injection of oligomycin (2 µM, port A), carbonyl cyanide-4 (trifluoromethoxy) phenylhydrazone (2 µM, port B), and antimycin A/rotenone (1 µM) in combination with Hoechst dye (10 µM, Port C) (all Sigma-Aldrich). The number of cells per well was quantified using a Biotek Cytation cellular imaging system, and the data were normalized using the Seahorse XF Imaging and Cell Counting software (both Agilent).

### 2.14 Fatty acid oxidation

Fatty acid oxidation in primary myoblasts isolated from Lal-/- and wild-type mice was determined as previously described with minor modifications [30]. In brief, 45,000 primary myoblasts were seeded in gelatin-coated T-25 flasks in the upright position and grown in growth medium until confluency. Thereafter, the cells were differentiated into myoblasts using differentiation medium for 3 days. The cells were rinsed with warm PBS and incubated for 1 h at 37 °C with 1 mL of substrate containing 100 µM palmitic acid, 0.4 µCi [1–^14^C]-palmitic acid (Hartmann Analytic, Braunschweig, Germany), 0.5 mM carnitine, and 0.3% BSA in DMEM (glucose-, pyruvate-, and glutamine-free; Thermo Fisher Scientific, Waltham, MA) in flasks sealed with a rubber stopper equipped with a hanging basket containing a filter paper saturated with 50 µL of 1 M NaOH. The reaction was stopped by the addition of 100 µL of 70% perchloric acid. The released ^14^CO_2_ was trapped at 37 °C for 2 h on a filter and quantitated by liquid scintillation counting. The results were normalized to the number of seeded cells.

### 2.15 Statistical analyses

The data were compiled and analyzed with GraphPad Prism 9 (GraphPad, Boston, MA). Group differences were calculated using unpaired Student's *t*-test and two-way ANOVA with Tukey post-hoc analysis. Data are presented as mean ± standard deviation (SD), with the following levels of statistical significance marked with asterisks: * p < 0.05, ** p ≤ 0.01, *** p ≤ 0.001.

## 3 Results

### 3.1 Impaired LAL activity does not affect proliferation of muscle cells

**Fig. 1:**
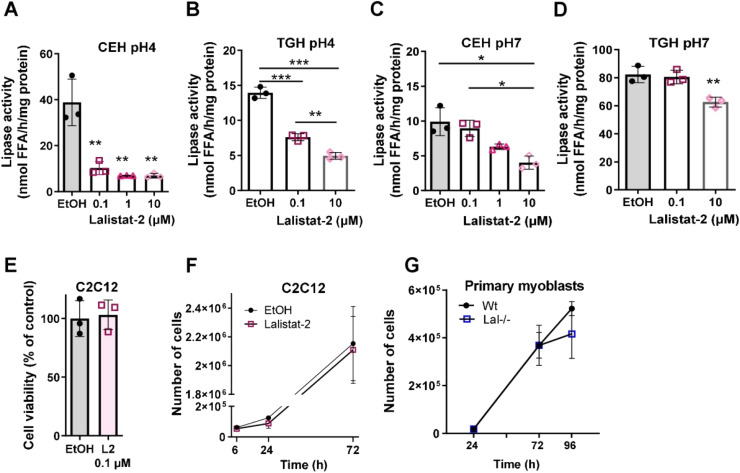
Defective LAL activity does not affect viability and proliferation of myoblasts. **(A, C)** Cholesteryl ester hydrolase (CEH) and **(B, D)** triacylglycerol hydrolase (TGH) activity at **(A, B)** acidic and **(C, D)** neutral pH in C2C12 cells treated with 0.1% EtOH (control) or different concentrations of Lalistat-2 (n=3). **(E)** Viability of differentiating C2C12 cells treated with 0.1 µM Lalistat-2. Proliferation rate of **(F)** C2C12 cells treated with 0.1 µM Lalistat-2 (n=3) and **(G)** primary myoblasts isolated from wild-type or Lal-/- mice (n=4). Data represent mean ± SD. *p < 0.05, **p ≤ 0.01, ***p ≤ 0.001. **(A-D, F, G)** Two-way ANOVA with Tukey post-hoc analysis. **(E)** Unpaired Student's *t*-test.

Maintenance of muscle mass is tightly regulated by the renewal of muscle cells through cellular processes such as proliferation and differentiation [31]. To investigate whether the loss of LAL activity affects the fate of muscle cells, we first incubated immortalized C2C12 myoblast cells with the LAL inhibitor Lalistat-2. Since this compound was shown to inhibit other enzymes as well, including the neutral lipases adipose triglyceride lipase, hormone-sensitive lipase, monoglyceride lipase, and neutral cholesterol ester hydrolase 1 [18], we incubated C2C12 cells with different concentrations of the inhibitor (0.1, 1, 10 µM) and determined CE and TG hydrolase activities at acidic and neutral pH values. Of the concentrations tested, 0.1 µM Lalistat-2 specifically reduced the activities of neutral CE and TG hydrolases at acidic but not at neutral pH conditions (Fig. 1A, B). Concentrations of Lalistat-2 above 0.1 µM were required to observe an inhibitory effect on neutral CE and TG hydrolase activities (Fig. 1C, D). These results confirmed the off-target effects of the inhibitor at higher concentrations also in C2C12 cells. Importantly, inhibition of LAL activity by 0.1 µM Lalistat-2 did not affect cell viability (Fig. 1E) or the proliferation rate as indicated by unaltered cell doubling of C2C12 cells (Fig. 1F). Based on these findings, we performed further experiments using Lalistat-2 at a concentration of 0.1 µM.

To further investigate SM development in the absence of *Lipa* gene expression (supplementary Fig. S1), we isolated primary myoblasts from the limb SM of Lal-/- mice. Comparable to C2C12 cells, genetic loss of LAL had no impact on the proliferation rate of primary myoblasts (Fig. 1G).

### 3.2 Loss of LAL activity in muscle cells affects neither myofiber formation nor the fiber phenotype

**Fig. 2:**
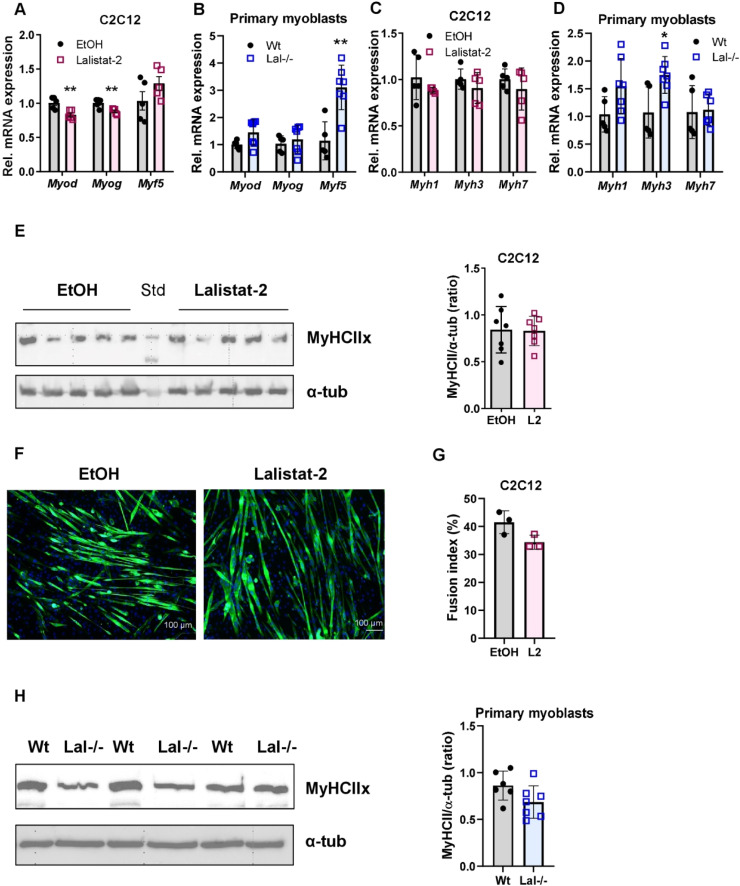
The loss of LAL activity in muscle cells does not impact myofiber formation. Relative mRNA expression of genes encoding differentiation markers of SM fibers relative to *cyclophilin A* expression as the reference gene in **(A)** C2C12 cells treated with EtOH (control) or 0.1 µM Lalistat-2 (n=5) and **(B)** primary myoblasts isolated from wild-type and Lal-/- mice (n=5–7). mRNA expression levels of genes encoding MyHC isoform genes (*Myh*) in **(C)** C2C12 cells (n=5) and **(D)** primary myoblasts isolated from wild-type and Lal-/- mice (n=5–7) relative *to cyclophilin A* expression as reference gene. **(E)** Representative Western blotting experiment of MyHCIIx protein expression and its densitometric quantification in C2C12 cells in the absence or presence of 0.1 µM Lalistat-2 (L2) treatment (n=6–7). **(F)** Representative images of immunofluorescence staining for MyHCIIx (green) in Lalistat-2-treated C2C12 cells differentiated for 6 days. Nuclei are stained with DAPI (blue). Scale bar, 100 µm. **(G)** The fusion index was calculated from 2–3 images from each of the 3 passages of C2C12 cells and represents the mean values of nuclei in the MyHCIIx-positive stained areas *versus* the total number of nuclei per image as a percentage. **(H)** Representative Western blotting experiment of MyHCIIx protein expression and its densitometric quantification in primary myoblasts isolated from wild-type or Lal-/- mice (n=6–7). Data represent mean ± SD. *p ≤ 0.05, **p ≤ 0.01. Unpaired Student's *t*-test.

Next, we determined the impact of LAL on myocyte differentiation. To this end, we treated confluent C2C12 cells with 0.1 µM Lalistat-2 for 6 days and measured the mRNA expression of the main myofiber differentiation markers. mRNA expression of myogenin (*Myog*) and myoblast determination protein 1 (*Myod*) was slightly reduced, whereas myogenic factor 5 (*Myf5*) remained unaltered (Fig. 2A). In primary myoblasts isolated from wild-type and Lal-/- SM, gene expression of *Myog* and *Myog* was comparable, whereas *Myf5* was even increased in Lal-/- cells (Fig. 2B), indicating that LAL deficiency or inhibition in the SM was not associated with impaired myofiber formation.

We have previously observed an increase in the expression of MyHCI (encoded by *Myh7*), specific for oxidative, slowly contracting fibers [5], in Lal-/- SM. In contrast, *Myh7* gene expression was comparable between Lalistat-2-treated and control C2C12 cells (Fig. 2C). Furthermore, the mRNA expression of *Myh3* (specific for developing fibers) and *Myh1* (a marker of fast glycolytic fibers) (Fig. 2C) as well as the protein expression of MyHCIIx (the predominant isoform of MyHC in the SM cell line) were unchanged in Lalistat-2-treated C2C12 cells (Fig. 2E). These data were corroborated by comparable MyHCIIx immunofluorescence staining and quantification of the fusion index (Fig. 2F, G). In primary myoblasts isolated from either Lal-/- or control SM (Fig. 2D), we observed a slightly upregulated mRNA expression of *Myh3* but comparable *Myh1* and *Myh7* mRNA (Fig. 2D) and MyHCIIx protein expression (Fig. 2H). These conflicting patterns of MyHC expression in the different models studied suggested that the SM phenotype observed in Lal-/- mice may be a consequence of systemic alterations and not directly caused by the loss of LAL in the SM.

### 3.3 Loss of LAL activity in muscle cells leads to lipid accumulation

**Fig. 3:**
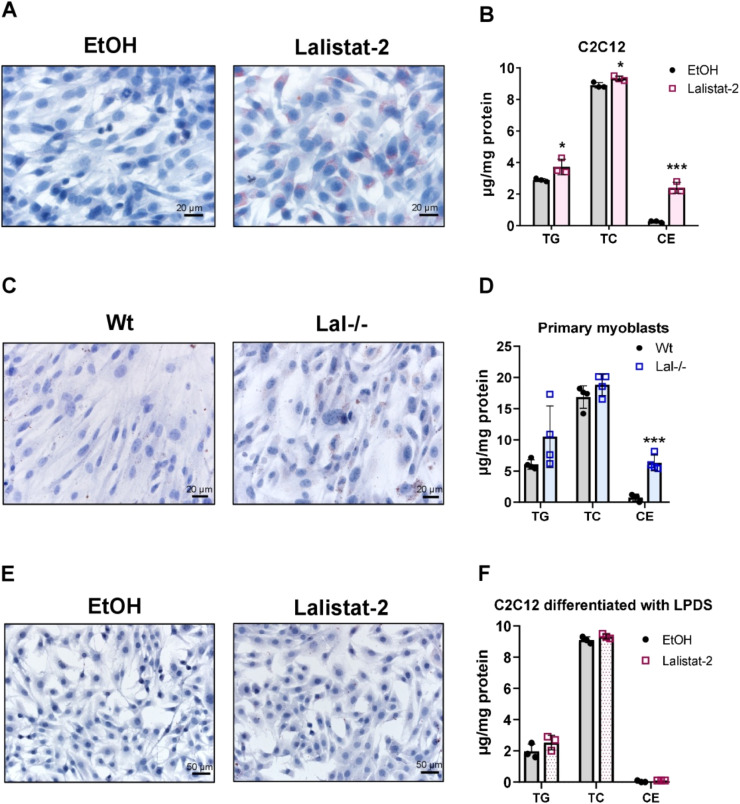
Inhibition of LAL with Lalistat-2 in C2C12 cells results in lipid accumulation. **(A)** Representative images of ORO-stained proliferating C2C12 cells cultivated in DMEM (25 mM glucose) plus FBS and treated with EtOH (control) or 0.1 µM Lalistat-2. **(B)** Biochemical analyses of lipid concentrations (triacylglycerol (TG), total cholesterol (TC), cholesteryl ester (CE)) (n=3). Magnification, 40X; scale bar, 20 µm. **(C)** Representative images of ORO-stained primary myoblasts isolated from wild-type or Lal-/- mice and differentiated in DMEM (25 mM glucose) plus horse serum. **(D)** Lipid quantification of primary myoblasts (n=4). Magnification, 40X; scale bar, 20 µm. **(E)** Representative images of ORO-stained C2C12 cells differentiated in LPDS-containing DMEM (25 mM glucose) and treated with 0.1 µM Lalistat-2 for 7 days. **(F)** Lipid quantification (n=3). Magnification, 20X; scale bar, 50 µm. Data represent mean ± SD *p ≤ 0.05, ***p ≤ 0.001. Unpaired Student's *t*-test.

Since LAL is responsible for the degradation of CE and TG in lysosomes, we next analyzed muscle cell lipids. Treatment with 0.1 µM Lalistat-2 resulted in lipid accumulation in proliferating C2C12 cells, as evidenced by oil red O (ORO) staining (Fig. 3A). A quantitative analysis of lipids revealed increased concentrations of TG, total cholesterol (TC), and particularly CE (Fig. 3B). Similarly, primary myoblasts isolated from Lal-/- mice exhibited increased lipid accumulation as visualized by ORO staining (Fig. 3C) with elevated CE concentrations, whereas the TG and TC levels only showed a trend toward higher values (Fig. 3D). Of note, Lal-/- mice have markedly lower levels of TG-rich very low-density lipoproteins in the fasted state [32]. To simulate this condition *in vitro*, we cultivated Lalistat-2-treated C2C12 cells in DMEM containing lipoprotein-deficient serum (LPDS) instead of horse serum. This culture condition resulted in no visible ORO staining (Fig. 3E) and unaltered lipid parameters (Fig. 3F). However, the viability of Lalistat-2-treated cells cultured in LPDS-containing medium was reduced (supplementary Fig. S2). In summary, the genetic ablation or pharmacological blockade of LAL activity is associated with an increase in CE (and TG) levels in muscle cells, provided that the cells have access to a sufficient amount of lipids.

### 3.4 Reduced abundance of α-ketoglutarate and fumarate in primary Lal-/- myoblasts

**Fig. 4:**
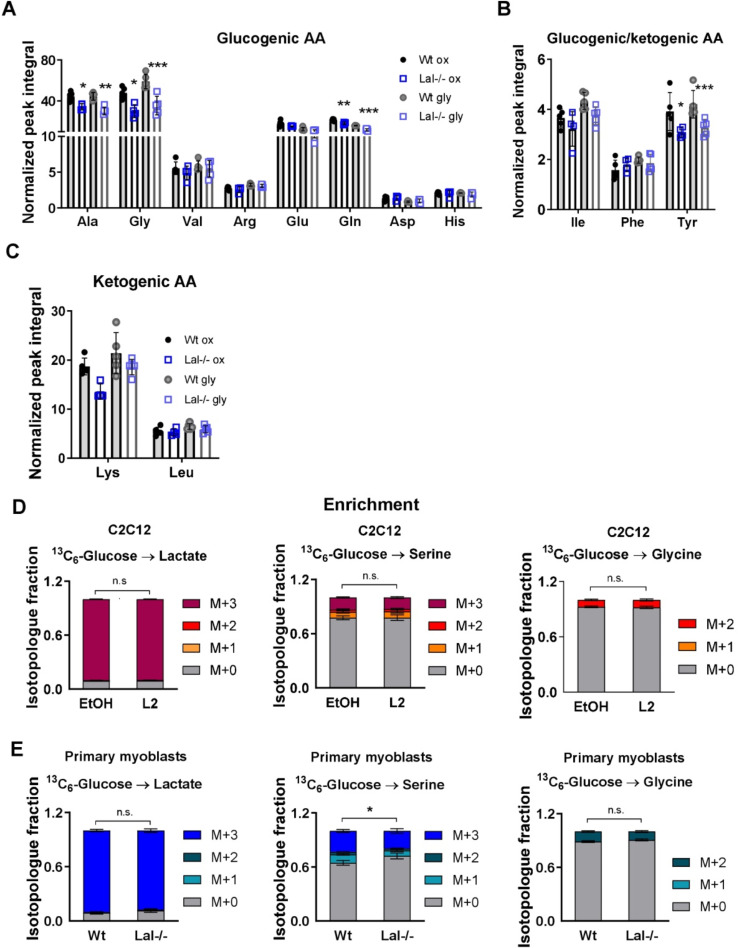

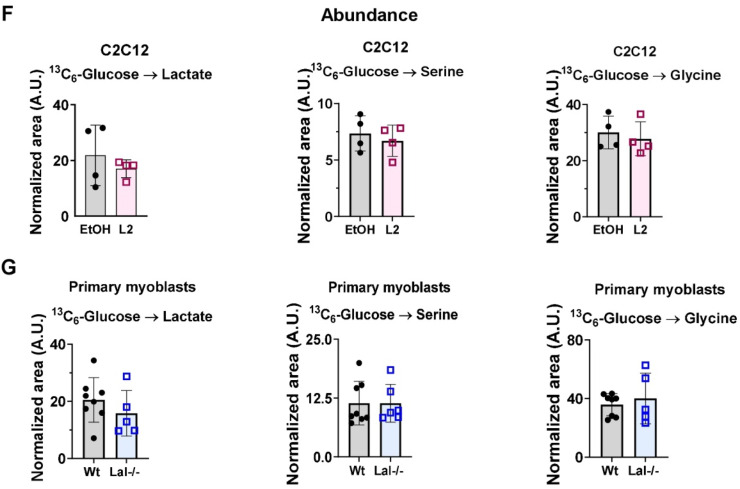
Loss of LAL affects amino acid concentrations in primary myoblasts. Individual **(A)** glucogenic, **(B)** glucogenic/ketogenic, and **(C)** ketogenic amino acid concentrations quantified by NMR. The data are presented as normalized peak integral enriched in oxidative (ox) or glycolytic (gly) segments of gastrocnemius from 12–17-week-old male wild-type and Lal-/- mice, with the mean ± SD (n=4–5) values indicated. Enrichment of lactate, serine, and glycine isotopologues after administration of uniformly labeled [^13^C_6_]-glucose to **(D)** C2C12 cells treated with EtOH or 0.1 µM Lalistat-2 (L2) and **(E)** primary myoblasts isolated from wild-type or Lal-/- mice. M+0 denotes unlabeled metabolites, and M+1, M+2, and M+3 contain one, two, or three ^13^C, respectively. Total abundance (normalized to protein and internal standard) of lactate, serine, and glycine in **(F)** C2C12 cells and **(G)** primary myoblasts loaded with [^13^C_6_]-glucose. Data represent mean ± SD. *p ≤0.05, **p ≤ 0.01, ***p ≤ 0.001; * in **E** indicates the difference between wild-type and Lal-/- cells in the M+0 isotopologue fraction. **(A-C, F, G)** Unpaired Student's *t*-test for the comparison of wild-type and Lal-/- samples of either ox or gly segments. **(D, E)** Two-way ANOVA with Tukey post-hoc analysis.

The observed alteration in lipid metabolism in Lalistat-2-treated C2C12 cells and primary Lal-/- myoblasts, as well as the increase in glucose uptake in Lal-/- SM *in vivo* [5], prompted us to investigate carbohydrate metabolism in the cellular models of genetic loss and pharmacological inhibition of LAL. Metabolomic analysis by nuclear magnetic resonance (NMR) revealed that glycine was one of the most significantly reduced amino acids in both the oxidative and glycolytic segments of gastrocnemius in Lal-/- mice (Fig. 4A-C). Analyses of tricarboxylic acid (TCA) cycle metabolites showed a slight reduction in succinate (supplementary Fig. S3A), but no difference in fumarate content (supplementary Fig. S3B) in the oxidative part from Lal-/- gastrocnemius. Therefore, we next employed stable isotope tracing experiments, in which we assessed the enrichment and abundance of glycolysis products and TCA cycle metabolites *in vitro* and *ex vivo* utilizing uniformly ^13^C-labeled (^13^C_6_-)glucose. A scheme of the possible labeling patterns of intermediates after the addition of [^13^C_6_]-glucose is shown in supplementary Fig. S4A.

First, we measured the ^13^C enrichment in lactate and serine/glycine, a glycolytic branching pathway. Neither pharmacological inhibition nor genetic loss of LAL resulted in an increased contribution of [^13^C_6_]-glucose to lactate or serine/glycine (Fig. 4D-E), nor did it lead to changes in the total abundance of lactate, serine, and glycine (Fig. 4F-G). It is noteworthy that primary myoblasts isolated from Lal-/- mice exhibited a slightly elevated unlabeled (M+0) serine fraction compared to myoblasts derived from wild-type mice (Fig. 4E). In contrast, the serine isotopologues were comparable between C2C12 cells treated with EtOH or Lalistat-2 (Fig. 4D). These results collectively suggested that glycolysis is not influenced by LAL activity in primary myoblasts or C2C12 cells.

**Fig. 5:**
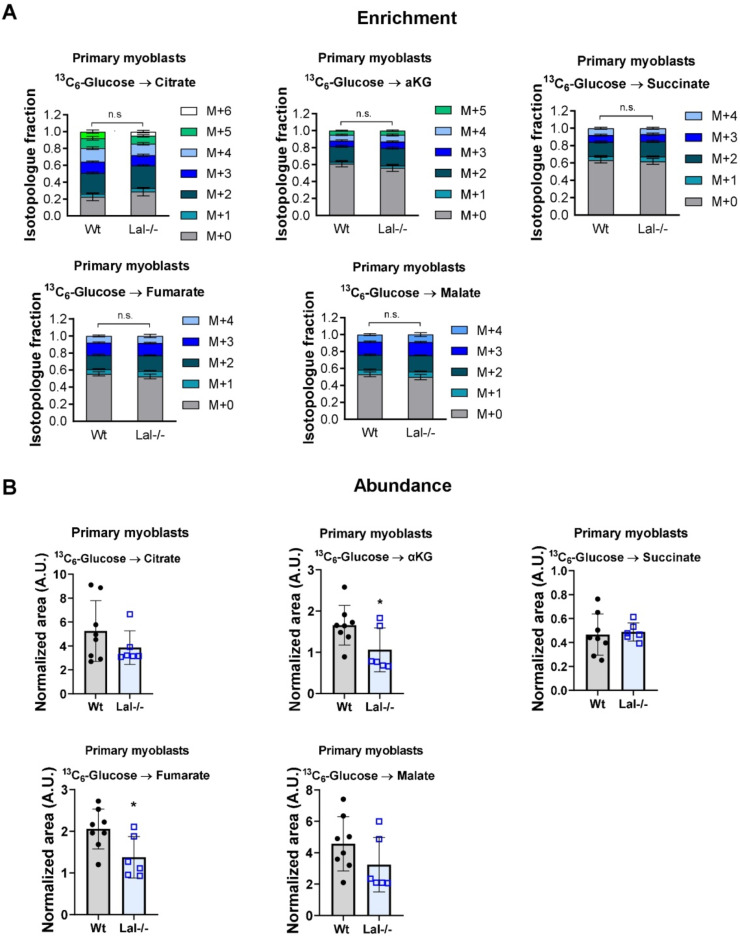
Reduced overall abundance of α-ketoglutarate and fumarate in primary Lal-/- myoblasts. **(A)** Enrichment of TCA cycle metabolite isotopologues after administration of uniformly labeled [^13^C_6_]-glucose to primary myoblasts isolated from wild-type or Lal-/- mice. M+0 denotes unlabeled metabolites, and M+1, M+2, M+3, M+4, M+5, and M+6 contain one, two, three, four, five or six ^13^C, respectively. **(B)** Total abundance (normalized to protein and internal standard) of TCA cycle metabolites in primary myoblasts loaded with [^13^C_6_]-glucose. αKG, α-ketoglutarate. Data represent mean ± SD. **(A)** Two-way ANOVA with Tukey post-hoc analysis. *p ≤ 0.05. **(B)** Unpaired Student's *t*-test.

Furthermore, we evaluated the enrichments and abundances of TCA cycle metabolites. [^13^C_6_]-glucose tracing in C2C12 cells showed the expected transfer of two carbons (M+2) from glucose to citrate via acetyl-CoA, with further transfer to α-ketoglutarate and fumarate, and higher isotopologues from consecutive TCA cycles. However, we did not detect any changes in the ^13^C label enrichments in TCA cycle intermediates (supplementary Fig. S4B, C). Similarly, primary myoblasts from wild-type and Lal-/- mice showed no difference in TCA cycle labeling (Fig. 5A). However, Lal-/- myoblasts exhibited a decrease in the total abundance of α-ketoglutarate and fumarate, along with a tendency toward reduced citrate abundance (Fig. 5B), indicating a reduction in the TCA cycle with less vailability of energy substrates to support bioenergetics in these cells.

### 3.5 Defective LAL activity is not associated with mitochondrial dysfunction in muscle cells

**Fig. 6:**
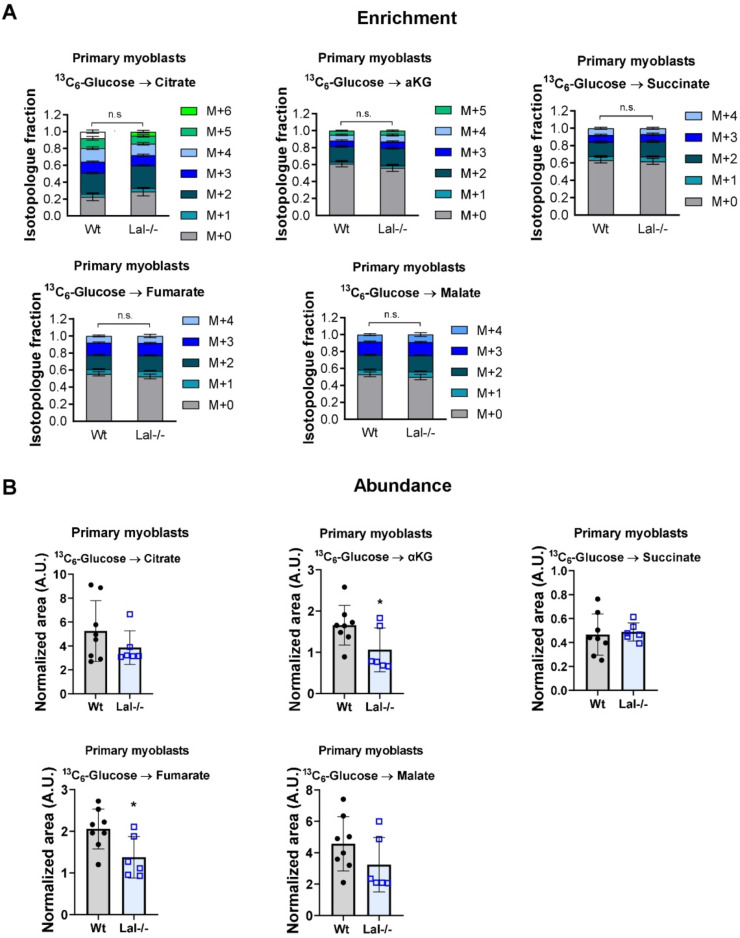
Loss or inhibition of LAL activity does not affect mitochondrial function. **(A)** Oxygen consumption rate (OCR) in C2C12 cells treated with EtOH (control) or 0.1 µM Lalistat-2, normalized to cell number. Arrows indicate the addition of the mitochondrial inhibitor oligomycin, the uncoupler carbonyl cyanide-4 (trifluoromethoxy) phenylhydrazone (FCCP), and the complex I and III inhibitors rotenone and antimycin A. Data represent mean (n=6) ± SD. **(B)** Oxidation of [1–^14^C]palmitic acid in primary myoblasts isolated from wild-type and Lal-/- mice (n=3–4). Data represent mean ± SD. Unpaired Student's *t*-test.

We have recently demonstrated that the oxidative capacity and ATP concentration are decreased in SM from Lal-/- mice [5]. In Lalistat-2-treated C2C12 cells, however, we were unable to confirm defective mitochondrial functions, as evidenced by the unchanged oxygen consumption rate (OCR) (Fig. 6A) and extracellular acidification rate (ECAR) (supplementary Fig. S5).

Given the findings in the permeabilized muscle fibers from Lal-/- mice, we finally measured mitochondrial fatty acid oxidation. However, primary myoblasts isolated from Lal-/- or wild-type SM revealed comparable amounts of the released ^14^CO_2_ radioactivity captured by the filter paper (Fig. 6B). Consequently, both our *in vitro* and *ex vivo* models demonstrated that inhibition or loss of LAL activity had no effect on mitochondrial function.

## 4 Discussion

Despite a normal protein turnover, Lal-/- mice exhibit reduced SM mass and size [5] and show a tendency toward decreased quadriceps mass as early as 4 weeks of age [21]. We also observed elevated SM CE levels and an impaired systemic metabolic balance, especially during fasting [5]. Proteomic analysis of gastrocnemius revealed an upregulation of proteins linked to fiber type transition, including increased MyHCI expression. Gene ontology analysis indicated a reduction in mitochondrial function and impaired oxidative phosphorylation [5]. However, it remained unclear whether these changes were a direct consequence of loss of LAL specifically in the SM or caused by systemic perturbations in the metabolic and inflammatory homeostasis due to enzyme deficiency in muscular tissues. In this study, we investigated the consequences of genetic loss and pharmacological inhibition of LAL in muscle cells on SM formation and metabolism. Our findings suggest that impaired LAL activity in muscle cells leads to lipid accumulation, particularly in CE, indicating a potential role for LAL in lipid degradation also in SM. Nevertheless, myofiber formation and mitochondrial functions were unaffected, suggesting a minor impact of LAL activity on SM growth and muscle energy supply.

The experiments in the present study were performed using primary myoblasts isolated from Lal-/- mice and/or C2C12 cells treated with Lalistat-2 at a concentration of 0.1 µM. Higher concentrations of Lalistat-2 inhibited also neutral CE and TG hydrolases, a finding consistent with previous observations in other cell types [18]. Despite decreased IGFBP-5 protein expression in Lal-/- SM [5], indicative of impaired SM differentiation [33], the expression levels of the myofiber differentiation markers *Myod* and *Myog* were only slightly reduced in Lalistat-2-treated C2C12 cells. Moreover, protein expression of the major isoform of MyHC was unaffected. Notably, 2-day-old Lal-/- mice also showed no difference in SM mass (Kuentzel et al, personal communication). In contrast, the results from primary Lal-/- myoblasts were inconsistent, with an upregulation of the myofiber differentiation marker *Myf5* and myosin heavy chain-embryonic *Myh3*. The fact that mRNA expression of other differentiation markers (*i.e., Myod, Myog*, and the myosin isoforms *Myh1* and *Myh7*) and MHCIIx protein expression were comparable between the genotypes suggests that the differentiation process as such is normal in both genotypes.

Genetic loss and pharmacological inhibition of LAL in muscle cells led to an accumulation of lipids, particularly CE. However, when C2C12 cells were allowed to differentiate in medium containing LPDS instead of horse serum, thereby mimicking the reduced plasma concentrations of very low-density lipoproteins observed in Lal-/- mice [32], Lalistat-2 treatment failed to affect cellular lipid content. In addition, the diminished viability of C2C12 cells in LPDS during proliferation indicate that lipids and functional LAL activity are indispensable for optimal muscle cell function and growth. The increase in glucose uptake of SM in Lal-/- mice [5,32] may reflect compensatory mechanisms in response to altered lipid metabolism. However, it is important to mention that elevated glucose uptake does not necessarily translate into normal metabolic function. The cellular inability to efficiently utilize the glucose may still persist, particularly in the presence of underlying metabolic derangements and altered mitochondrial function. The remarkable reduction of lactate [32] and glycine concentrations in the gastrocnemius of Lal-/- mice, however, were not recapitulated in muscle cells *in vitro*, where the contributions of [^13^C_6_]-glucose to lactate and serine/glycine were comparable to control cells. This discrepancy suggests that, while overall metabolite concentrations may be altered *in vivo*, the flux of labeled glucose to specific metabolites is not affected by the loss or inhibition of LAL in muscle cells *per se*. Further analysis of TCA cycle metabolites revealed intriguing differences between the various experimental approaches. The decrease in the overall abundance of α-ketoglutarate and fumarate in primary Lal-/- myoblasts suggests perturbations in TCA cycle intermediary metabolism related to LAL-D. However, it is worth noting that data from metabolomic analysis of Lal-/- SM did not consistently support these findings. For instance, lower concentrations of succinate were observed in oxidative fibers of Lal-/- gastrocnemius, highlighting the complexity of metabolic alterations in LAL-D.

In contrast to the findings in Lal-/- mice, in which we observed a decrease in mitochondrial function and an increase in the expression of genes and proteins linked to slow oxidative fibers [5], we were unable to reproduce the results on defective mitochondrial function or changes in fiber type composition in Lalistat-2-treated C2C12 cells and primary Lal-/- myoblasts. The discrepancies observed between the *in vivo* and *in vitro* models might be attributed to several factors. For instance, in our previous study, using permeabilized fibers freshly isolated from the gastrocnemius muscle, any mitochondrial damage that had occurred *in vivo* could not be reversed within the short experimental timeframe, despite the availability of substrates. This contrasts with the *in vitro* conditions of the present study, where cell proliferation and differentiation occurred in the presence of abundant nutrients, possibly preventing mitochondrial damage and leading to opposite results. Thus, the *in vivo* environment exposes mitochondria to chronic stressors and damage that accumulate over time, which may not be rapidly mitigated by substrate availability alone, whereas the controlled *in vitro* conditions allow for optimal mitochondrial function and repair. Indeed, adult Lal-/- mice lack white adipose tissue, leading to drastic dyslipidemia with a systemic reduction in TG availability and a potential impact on SM. Furthermore, the rapid glucose consumption of various organs in these animals [11,32,34] very likely contributes to the discrepancies observed. In contrast, cells grown *in vitro* are exposed to a glucose-rich environment (25 mM) and survive poorly in a low-glucose medium (5.6 mM). In addition, systemic inflammation profoundly influences the SM phenotype, as evidenced in various pathological conditions such as cachexia [[35], [36], [37]], diabetes [38,39], or chronic obstructive pulmonary disease [40,41]. The consistent elevation of pro-inflammatory cytokines and the substantial presence of macrophages in multiple organs indicate systemic inflammation in Lal-/- mice, a phenomenon that is challenging to replicate in cell culture experiments. Future studies should aim to elucidate the mechanisms underlying these metabolic alterations.

In conclusion, while inhibition of LAL with Lalistat-2 in C2C12 cells or loss of LAL in primary myoblasts from Lal-/- mice affected intracellular lipid parameters and metabolism, it had no apparent effects on overall myofiber formation or mitochondrial functions. However, the present cell experiments provide evidence that the changes observed in Lal-/- mice are the consequence of a global loss of the enzyme, which primarily affects the liver, small intestine, and macrophages and causes systemic inflammation. Although LAL plays a pivotal role in lipid metabolism of SM, it has only a minor influence on the processes of muscle differentiation and cellular energy production.

## Glossary

**ANOVA:** analysis of variance

**ATP:** adenosine triphosphate

**CE:** cholesteryl ester

**CEH:** CE hydrolase

**DMEM:** Dulbecco's Modified Eagle's Medium

**FC:** free cholesterol

**ECAR:** extracellular acidification rate

**GAPDH:** glyceraldehyde 3-phosphate dehydrogenase

**GC – MS:** gas chromatography–mass spectrometry

**LAL:** lysosomal acid lipase

**Lal-/-:** LAL-deficient

**LAL-D:** lysosomal acid lipase deficiency

**LPDS:** lipoprotein-deficient serum

**MyHC:** myosin heavy chain

**MTT:** 3-[4,5-dimethylthiazol-2-yl]-2,5-diphenyltetrazolium bromide

**NMR:** nuclear magnetic resonance

**OCR:** oxygen consumption rate

**ORO:** Oil Red O

**SD:** standard deviation

**SM:** skeletal muscle

**TBDMS**: N-(tert-butyldimethylsilyl)-N-methyl-trifluoroacetamide

**TC:** total cholesterol

**TCA:** tricarboxylic acid cycle

**TG:** triacylglycerol

**TGH:** TG hydrolase

## Funding

This work was supported by the Austrian Science Fund (FWF) (SFB 10.55776/F73, DK-MCD 10.55776/W1226, 10.55776/P32400, 10.55776/P33508), the Integrative Metabolism Research Center Graz, the Austrian Infrastructure Program 2016/2017, the flagship project “VascHealth” and the PhD program “Molecular Medicine” of the Medical University of Graz, the Province of Styria, and the City of Graz. For open access purposes, the authors have applied a CC BY public copyright license to any author accepted manuscript version arising from this submission.

## Consent for publication

All authors consent to publication.

## Ethics approval and consent to participate

There were no human data analyzed. All experimental procedures with mice were conducted in accordance with the European Directive 2010/63/EU and approved by the Austrian Federal Ministry of Education, Science and Research, Vienna, Austria (2022-0.920.281).

## CRediT authorship contribution statement

**Alena Akhmetshina:** Writing – original draft, Methodology, Investigation, Formal analysis, Data curation, Conceptualization. **Laszlo Schooltink:** Writing – review & editing, Investigation, Formal analysis, Data curation. **Melina Amor:** Writing – review & editing, Investigation. **Katharina B. Kuentzel:** Writing – review & editing, Investigation, Formal analysis. **Silvia Rainer:** Investigation. **Ananya Nandy:** Writing – review & editing, Investigation. **Hansjoerg Habisch:** Writing – review & editing, Methodology, Investigation. **Tobias Madl:** Writing – review & editing, Resources, Methodology, Funding acquisition. **Elizabeth Rendina-Ruedy:** Writing – review & editing, Supervision, Resources. **Katharina Leithner:** Writing – review & editing, Resources, Methodology, Investigation, Funding acquisition, Formal analysis. **Nemanja Vujić:** Writing – review & editing, Investigation, Formal analysis, Conceptualization. **Dagmar Kratky:** Writing – review & editing, Resources, Project administration, Funding acquisition, Data curation, Conceptualization.

## Declaration of competing interest

The authors declare that no conflict of interest exists.

## Data Availability

Data will be made available on request.

## Appendix Supplementary materials

Image, application 1
