# Supplementary material for: Loss or inhibition of lysosomal acid lipase in vitro leads to cholesteryl ester accumulation without affecting muscle formation or mitochondrial function
Source: BBA Adv. 2024 Dec 25;7:100135. doi: 10.1016/j.bbadva.2024.100135 (PMC11745973; doi:10.1016/j.bbadva.2024.100135)
Supplement: Supplementary file 1 [file mmc1.pdf]

Supplementary Figures and Tables

Loss or inhibition of lysosomal acid lipase in vitro leads to cholesteryl ester accumulation without affecting muscle formation or mitochondrial function

Alena Akhmetshina, Laszlo Schooltink, Melina Amor, Katharina B. Kuentzel, Silvia Rainer, Ananya Nandy, Hansjoerg Habisch, Tobias Madl, Elizabeth Rendina-Ruedy, Katharina Leithner, Nemanja Vujić, Dagmar Kratky

Figure S1

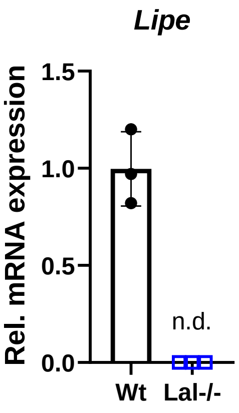

Figure S1. Confirming the efficient deletion of *Lipa* in primary Lal-/- myoblasts. *Lipa* gene expression during differentiation of primary myoblasts isolated from Wt and Lal-/- mice relative to *cyclophilin A* expression (n=3). Data represent mean ± SD. Unpaired Student's t-test. n.d., not detected.

Figure S2

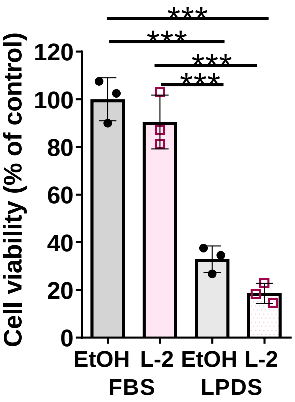

Figure S2. Reduced viability of C2C12 cell treated with Lalistat-2 in LPDS-containing medium. The viability of proliferating C2C12 cells treated with 0.1 µM Lalistat-2 (L2) in FBS or LPDS-containing medium presented in % of control (EtOH). Data represent mean ± SD (n=3). ***p ≤ 0.001. Two-way ANOVA with Tukey post-hoc analysis.

Figure S3

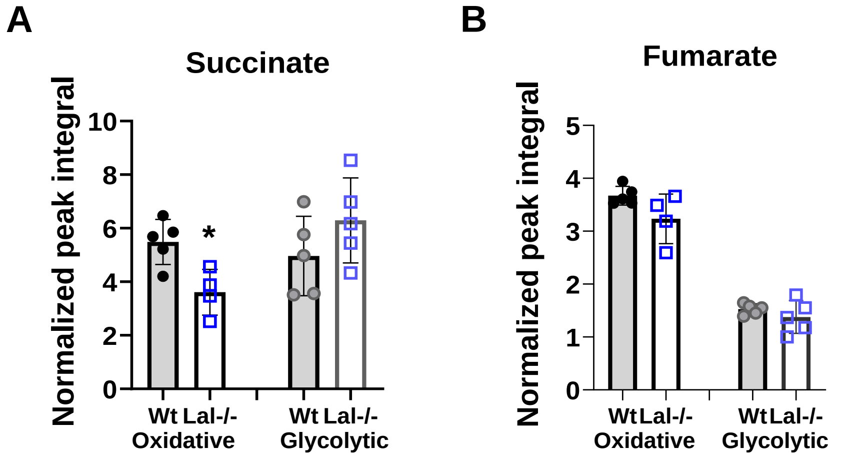

Figure S3. Altered succinate concentration in gastrocnemius of Lal-/- mice. (A) Succinate and **(B)** fumarate of 12–17-weeks old male Wt and Lal-/- mice were quantified by NMR. Data are presented as normalized peak integral enriched in oxidative or glycolytic segments of gastrocnemius from Wt and Lal-/- mice (n=4-5) as mean ± SD. *$p < 0.05$. Unpaired Student's t-test for the comparison of Wt and Lal-/- samples of either oxidative or glycolytic segments.

Figure S4

A

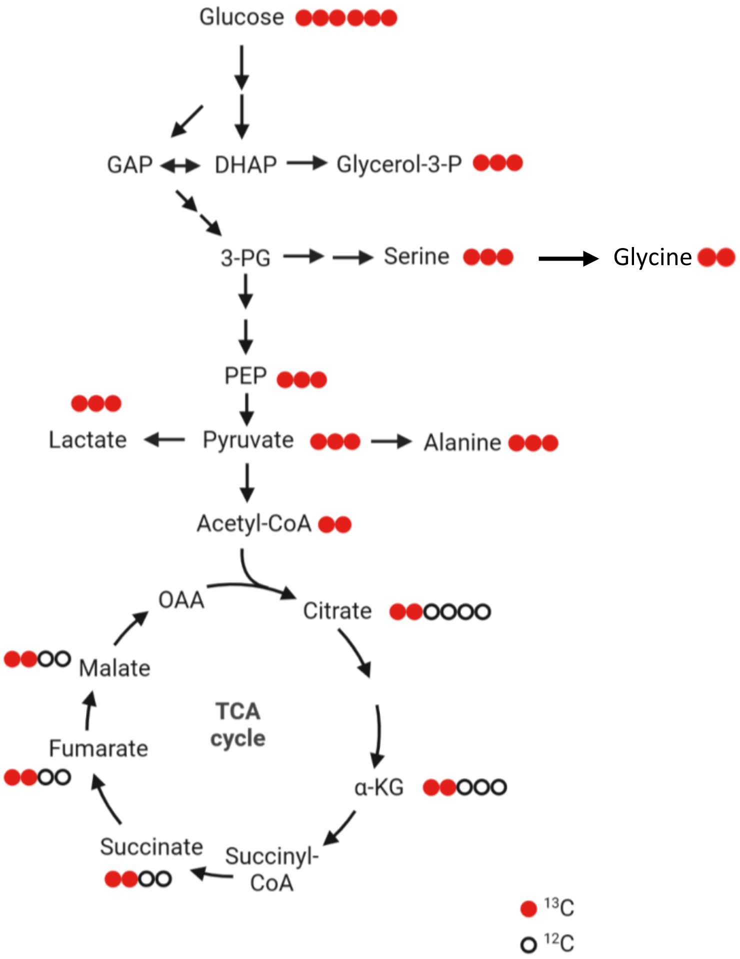

Glucose
GAP
DHAP
Glycerol-3-P
3-PG
Serine
Glycine
PEP
Lactate
Pyruvate
Alanine
Acetyl-CoA
OAA
Citrate
Malate
TCA cycle
Fumarate
α-KG
Succinate
Succinyl-CoA
13C
12C

B

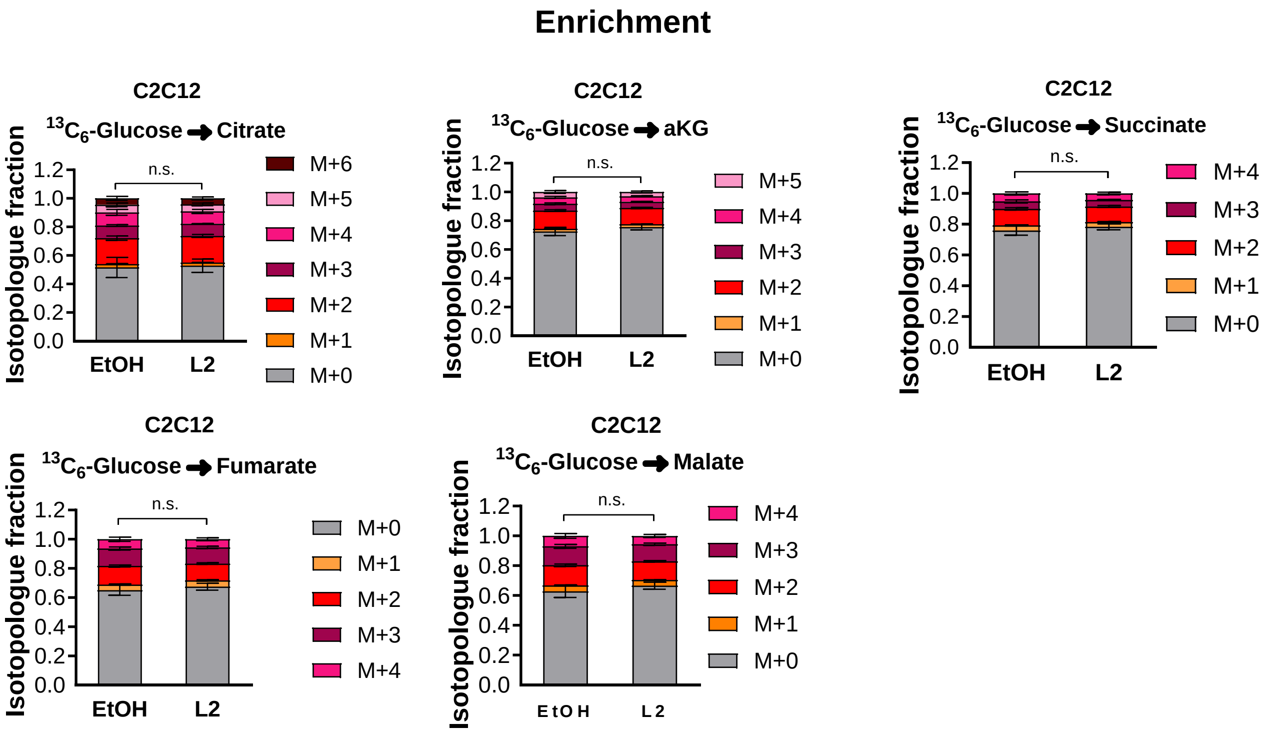

Enrichment
C2C12
13C6-Glucose → Citrate
Isotopologue fraction
n.s.
EtOH
L2
M+6
M+5
M+4
M+3
M+2
M+1
M+0
C2C12
13C6-Glucose → aKG
C2C12
13C6-Glucose → Succinate
C2C12
13C6-Glucose → Fumarate
C2C12
13C6-Glucose → Malate

C

Abundance

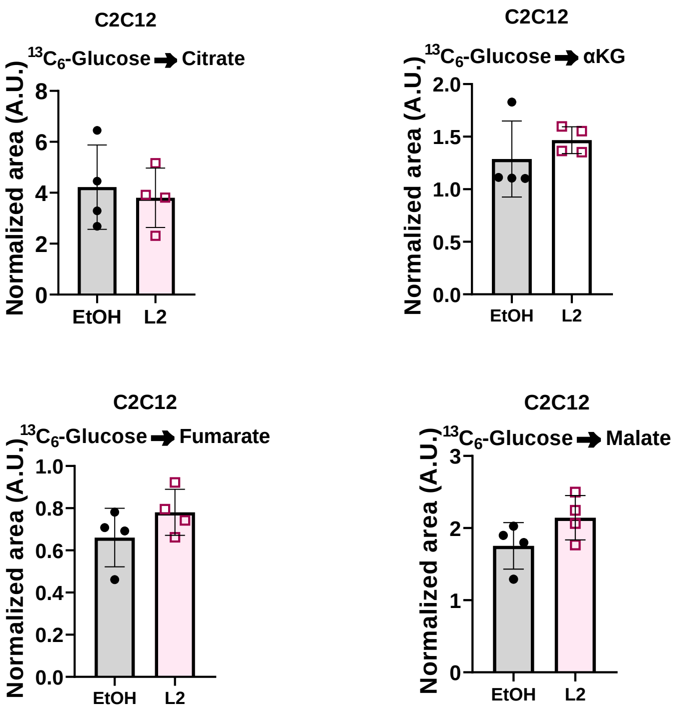

Figure S4. Tricarboxylic acid (TCA) cycle metabolites in C2C12 cells are unaffected by Lalistat-2 treatment. (A) Metabolic pathway for the conversion of glucose to serine/glycine or/and pyruvate. Filled circles represent $[^{13}C]$-labeled glucose. DHAP, dihydroxyacetone phosphate; GAP, glyceraldehyde 3-phosphate; 3-PG, 3-phosphoglyceric acid; OAA, oxaloacetic acid; TCA cycle, tricarboxylic acid cycle; PEP, phosphoenolpyruvate; αKG, α-ketoglutarate; PCK_2, phosphoenolpyruvate carboxykinase mitochondrial isoform. **(B)** Enrichment of TCA cycle metabolite isotopologues after administration of uniformly labeled $[^{13}C_6]$-glucose to C2C12 cells treated with EtOH (control) or 0.1 µM Lalistat-2 (L2). M+0 denotes unlabeled metabolites, and M+1, M+2, M+3, M+4, M+5, and M+6 contain one, two, three, four, five or six ^{13}C, respectively. **(C)** Total abundance (normalized to protein and internal standard) of TCA cycle metabolites in $[^{13}C_6]$-glucose-loaded C2C12 cells treated with EtOH or L2. Data represent mean ± SD. **(B)** Two-way ANOVA with Tukey post-hoc analysis. **(C)** Unpaired Student's t-test.

Figure S5

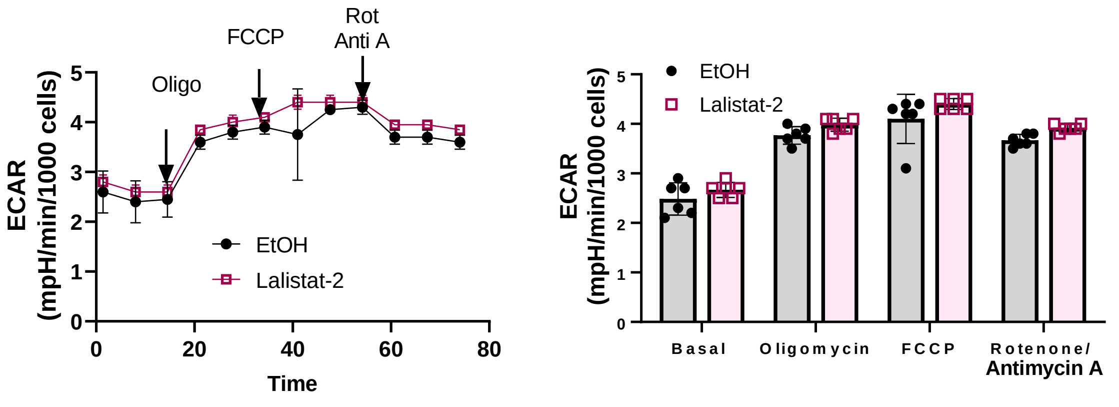

Figure S5. Inhibition of LAL does not affect extracellular acidification rates in C2C12 cells. Extracellular acidification rates (ECAR) during the treatment of C2C12 cells with EtOH (control) or 0.1 µM Lalistat-2 normalized to cell number. Arrows indicate the addition of the mitochondrial inhibitor oligomycin, the uncoupler carbonyl cyanide-4 (trifluoromethoxy) phenylhydrazone (FCCP), and the complex I and III inhibitors rotenone/antimycin A. Data represent mean ± SD. Unpaired Student's t-test.

Table S1. Primer sequences used for real-time PCR.

Gene	Forward sequence (5’-3’)	Reverse sequence (5’-3’)
Cyclophilin A	CCATCCAGCCATTCAGTCTT	TTCCAGGATTCATGTGCCAG
Lipa	GCTGGCTTTGATGTGTGGATG	ATGGTGCAGCCTTGAGAATGA
Myf5	GCCTTCGGAGCACACAAAG	TGACCTTCTTCAGGCGTCTAC
Myh1	CTCTTCCCGCTTTGGTAAGTT	CAGGAGCATTTCGATTAGATCCG
Myh3	CCAAAACCTACTGCTTTGTGGT	GGGTGGGTTCATGGCATACA
Myh7	AGACTGTCAACACTAAGAGGGT	TGCCCCAAAATGGATTCGGAT
Myod	CGGGACATAGACTTGACAGGC	TCGAAACACGGGTCATCATAGA
Myog	GAGACATCCCCCTATTTCTACCA	GCTCAGTCCGCTCATAGCC
